# Outbreaks Associated With Environmental and Undetermined Water Exposures — United States, 2011–2012

**DOI:** 10.15585/mmwr.mm6431a3

**Published:** 2015-08-14

**Authors:** Karlyn D. Beer, Julia W. Gargano, Virginia A. Roberts, Hannah E. Reses, Vincent R. Hill, Laurel E. Garrison, Preeta K. Kutty, Elizabeth D. Hilborn, Timothy J. Wade, Kathleen E. Fullerton, Jonathan S. Yoder

**Affiliations:** 1 Epidemic Intelligence Service, CDC; 2 Division of Foodborne, Waterborne, and Environmental Diseases, National Center for Emerging and Zoonotic Infectious Diseases, CDC; 3 Division of Bacterial Diseases, National Center for Immunization and Respiratory Diseases, CDC; 4 Environmental Protection Agency

Exposures to contaminated water can lead to waterborne disease outbreaks associated with various sources, including many that are classified and reported separately as drinking water† (1) or recreational water§ (2). Waterborne disease outbreaks can also involve a variety of other exposures (e.g., consuming water directly from backcountry or wilderness streams, or inhaling aerosols from cooling towers and ornamental fountains). Additionally, outbreaks might be epidemiologically linked to multiple water sources or may not have a specific water source implicated.

This report describes waterborne disease outbreaks associated with environmental and undetermined water exposures (combining and replacing the previously reported categories “water not intended for drinking,” “water of unknown intent,” and “other nonrecreational water”) (3,4), in which the first illness occurred in 2011 or 2012.¶ Outbreaks that were reported to the Waterborne Disease and Outbreak Surveillance System (http://www.cdc.gov/healthywater/surveillance/index.html) through the electronic National Outbreak Reporting System (http://www.cdc.gov/nors/about.html) as of October 30, 2014, were included. Data collected for each outbreak include the numbers of cases of illness, hospitalizations, and deaths; the suspected or confirmed etiologic agent; the implicated water source; and the setting of exposure.

During 2011–2012, public health officials from 11 states reported 18 outbreaks associated with environmental or undetermined water exposures, causing 280 cases of illness, 67 hospitalizations (24% of cases), and 10 deaths (Table). These 18 outbreaks included 15 legionellosis outbreaks that resulted in 254 cases and all 10 deaths. The legionellosis outbreaks occurred in hotels and motels (n = four), hospitals and healthcare facilities (n = three),** long-term–care facilities (n = three), an indoor workplace/office (n = one), a factory/industrial setting (n = one), a mobile home park (n = one), a resort (n = one), and a multi-use facility (n = one). Five legionellosis outbreaks had a known water source, including ornamental fountains (n = three), a cooling tower (n = one), and a storage tank (n = one). For 10 legionellosis outbreaks the water source was undetermined. Among these, one outbreak had multiple implicated sources (drinking water, spa, and cooling system), and the remaining nine had insufficient data to implicate a particular source. Five of the 10 deaths caused by *Legionella* were health care facility–associated, including two associated with long-term care facilities, two with hospitals, and one with an unknown type of health care facility. In addition to the 15 legionellosis outbreaks, three *Giardia intestinalis* outbreaks occurred, following drinking of untreated water directly from rivers or streams in outdoor settings.

Waterborne disease outbreaks not associated with drinking water or recreational water have been increasingly reported during the past 10 years. The increase is primarily associated with an increasing number of reported *Legionella* outbreaks, concomitant with the rise in *Legionella* outbreaks associated with drinking water systems (1) (http://www.cdc.gov/healthywater/surveillance/drinking-water-tables-figures.html). The variety of settings and water sources implicated in the *Legionella* outbreaks reported here highlights the complexity of *Legionella* control and mitigation in the built environment, particularly in settings where susceptible persons congregate, such as hospitals, long-term care facilities, and other healthcare settings (5). Outbreaks associated with untreated water sources highlight the importance of properly treating water for drinking in backcountry settings (http://www.cdc.gov/healthywater/drinking/travel/backcountry_water_treatment.html). Continued support for enhanced epidemiologic and environmental investigations of waterborne disease outbreaks would enable better classification of outbreaks with undetermined water exposures. Subsequently, closer examination of the reported outbreaks with emerging environmental and undetermined water exposures might reveal opportunities for detecting and preventing disease associated with the diverse water exposures encountered in everyday life.

## Figures and Tables

**TABLE:** Waterborne disease outbreaks associated with environmental and undetermined water exposures* (n = 18), by state or jurisdiction and month of first case onset — Waterborne Disease and Outbreak Surveillance System, United States, 2011–2012

Exposure state/ Jurisdiction	Month	Year	Etiology†	Predominant illness§	No. cases	No. hospitalizations¶	No. deaths^**^	Water source	Setting
Colorado	May	2011	*Giardia intestinalis*	AGI	2	0	0	River/Stream	Camp/Cabin
Florida	Oct	2011	*L. pneumophila* serogroup 1	ARI	3	3	1	Ornamental fountain	Mobile home park
Florida	Nov	2012	*L. pneumophila*	ARI	2	2	0	Undetermined	Hotel/Motel/Lodge/Inn
Idaho	Feb	2012	*Giardia intestinalis*	AGI	4	0	0	River/Stream	National forest
Illinois	Jul	2012	*L. pneumophila* serogroup 1	ARI	114	15	3	Ornamental fountain	Hotel/Motel/Lodge/Inn
Illinois	Aug	2012	*Legionella* suspected††	ARI	56	0	0	Ornamental fountain	Hotel/Motel/Lodge/Inn
Massachusetts	Sep	2011	*L. pneumophila* serogroup 1	ARI	2	2	0	Undetermined	Indoor Workplace/Office
New York	Jul	2011	*L. pneumophila* serogroup 1	ARI	5	4	1	Cooling tower	Hospital/Health care
New York	Jul	2011	*L. pneumophila* serogroup 1	ARI	2	2	1	Undetermined	Hospital/Health care
Ohio	Jan	2010§§	*L. pneumophila* serogroup 1	ARI	4	4	1	Undetermined	Hospital/Health care
Ohio	Jul	2011	*L. pneumophila* serogroup 1	ARI	3	3	1	Undetermined	Hospital/Health care
Ohio	Sep	2011	*L. pneumophila* serogroup 1	ARI	5	5	1	Undetermined	Other¶¶
Ohio	Feb	2012	*L. pneumophila* serogroup 1	ARI	8	8	2	Undetermined	Long-term care facility
Ohio	Nov	2012	*L. pneumophila* serogroup 1	ARI	7	3	0	Undetermined	Long-term care facility
Pennsylvania	Aug	2011	*L. pneumophila* serogroup 1	ARI	8	4	0	Undetermined	Long-term care facility
Pennsylvania	Jul	2012	*L. pneumophila* serogroup 1	ARI	34	11	0	Undetermined (Multiple)	Hotel/Motel/Lodge/Inn
Utah	Nov	2011	*Giardia intestinalis*	AGI	20	0	0	River/Stream	Public outdoor area
West Virginia	Jun	2011	*L. pneumophila* serogroup 1	ARI	3	3	0	Undetermined	Resort
Wisconsin	May	2012	*L. pneumophila* serogroup 1	ARI	2	2	0	Storage tank	Factory/Industrial facility

**Abbreviations:** AGI = acute gastrointestinal illness; ARI = acute respiratory illness; *L. pneumophila* = *Legionella pneumophila*; other = undefined, illnesses, conditions, or symptoms that cannot be categorized as gastrointestinal, respiratory, ear-related, eye-related, skin-related, neurologic, hepatitis, or caused by leptospirosis.

* The environmental and undetermined category includes outbreaks not associated with drinking water systems (public, private or bottled water) or recreational water venues (e.g., swimming pools, lakes), and includes outbreaks epidemiologically linked to multiple water sources and outbreaks without a specific implicated water source.

† Etiologies listed are confirmed, unless indicated “suspected”; for multiple-etiology outbreaks, etiologies are listed in alphabetical order.

§ The category of illness reported by =50% of ill respondents; all legionellosis outbreaks were categorized as ARI.

¶ Value was set to “missing” in reports where zero hospitalizations were reported and the number of people for whom information was available was also zero.

†† Outbreak of Pontiac Fever; Legionella pneumophila serogroup 1 found in water sample.

§§ The first case of illness in this outbreak occurred before 2011, but the outbreak was reported later and not previously described in a surveillance report.

¶¶ Multiuse facility serving individuals with disabilities.

## Footnotes

† Drinking water, also called potable water, is water for human consumption (e.g., drinking, bathing, showering, hand washing, teeth brushing, food preparation, dishwashing, maintaining oral hygiene) and includes water collected, treated, stored, or distributed in public and individual water systems, as well as bottled water.

§ Recreational water includes water in venues that are treated (e.g., pools, hot tubs, spas) or untreated (e.g., lakes, oceans).

¶ One previously unreported outbreak with first case onset date in 2010 is reported in the Table, but not summarized in text.

** Characteristics of hospitals and health care facilities (e.g., inpatient vs. outpatient) are not systematically collected in the National Outbreak Reporting System (NORS); however, long-term care facility settings are specified in NORS and might be reported as a subset of hospitals and health care facilities. Of three health care–associated legionellosis outbreaks, two were reported to have occurred in hospitals with inpatient facilities; the third occurred in an unspecified type of health care facility.
